# The TLR7 agonist induces tumor regression both by promoting CD4^+^T cells proliferation and by reversing T regulatory cell-mediated suppression via dendritic cells

**DOI:** 10.18632/oncotarget.2757

**Published:** 2014-12-15

**Authors:** Chenchen Wang, Quan Zhou, Xiaofeng Wang, Xiongyan Wu, Xuehua Chen, Jianfang Li, Zhenggang Zhu, Bingya Liu, Liping Su

**Affiliations:** ^1^ Shanghai Key Laboratory of Gastric Neoplasms, Shanghai Institute of Digestive Surgery, Department of Surgery, Ruijin Hospital, Shanghai Jiao Tong University School of Medicine, Shanghai 200025, People's Republic of China

**Keywords:** TLR7, Treg cells, Immune suppression, Anti-tumor effect

## Abstract

Treg-induced immunosuppression is now recognized as a key element in enabling tumors to escape immune-mediated destruction. Although topical TLR7 therapies such as imiquimod have been proved successful in the treatment of dermatological malignancy and a number of conditions beyond the FDA-approved indications, the mechanism behind the effect of TLR7 on effector T cell and Treg cell function in cancer immunosurveillance is still not well understood. Here, we found that Loxoribin, one of the TLR7 ligands, could inhibit tumor growth in xenograft models of colon cancer and lung cancer, and these anti-tumor effects of Loxoribin were mediated by promoting CD4^+^T cell proliferation and reversing Treg-mediated suppression via dendritic cells (DCs). However, deprivation of IL-6 using a neutralizing antibody abrogated the ability of Loxoribin-treated DCs, which reversed the Treg cell-mediated suppression. Furthermore, adoptive transfer of Loxoribin-treated DCs inhibited the tumor growth *in vivo*. Thus, this study links TLR7 signaling to the functional control of effector T cells and Treg cells and identifies Loxoribin as a new therapeutic strategy in cancer treatment, which may offer new opportunities to improve the outcome of cancer immunotherapy.

## INTRODUCTION

Tumor-induced immunosuppression is now recognized as a key element in enabling tumors to escape immune-mediated destruction [1, 2]. It is now evident that immune responses in cancer are negatively regulated by immunosuppressive cells, mainly T regulatory cells (Tregs) and myeloid-derived suppressor cells (MDSCs) [3–5]. They are largely responsible for inhibiting host T-cell activity against tumor associated antigens and consequently impair the effectiveness of anti-cancer immunotherapeutic approaches. Therefore, approaches aiming to reduce the deleterious effects of these immunosuppressive cells may increase the success of various immunotherapeutic modalities in cancer patients.

Increased proportions of CD4^+^CD25^+^Treg cells have been observed in patients with different types of cancer; thus, how to eliminate the suppressive function of Treg cells is a key question in cancer immunotherapy [6, 7]. Many strategies have been explored to block the suppressive function of Tregs in cancer patients [8, 9]. Toll-like receptors (TLRs) are pathogen-associated molecular patterns (PAMPs) that participate in the regulation of immune responses [10–12]. They are broadly expressed in various immunocytes, especially in innate cells such as dendritic cells (DCs) and macrophages [13, 14]. Recent studies have demonstrated that TLRs can directly or indirectly regulate the suppressive activity of Treg cells [10, 15]. In 2003, Chandrashekhar Pasare and Ruslan Medzhitov first demonstrated that LPS, the ligand of TLR4, could interact with TLR4 that was expressed on the surface of DCs and subsequently activated MyD88 signal pathway, thus releasing the suppressive function of Tregs on conventional CD4^+^T cells [16–19]. Pam3Cys-SK4, the ligand of TLR2, could directly function on Tregs, and made the Tregs lose the suppressive function [20–22]. Recently, Peng and his colleagues found that Poly(G), the ligand of TLR8, could directly function on Tregs and decrease their suppressive activity on CD4^+^T cells [23]. Thus, TLRs ligands may be potential immunotherapeutic reagents to cancer patients. Actually, imiquimod, a TLR7/TLR8 ligand, is a widely used topical immune response modifier, which is a U.S. Food and Drug Administration (FDA)-approved treatment for external genital warts, actinic keratoses (AKs), and superficial basal cell carcinomas (sBCCs) [24, 25]. By triggering cytokine production such as IFN-γ, imiquimod enhances the ability of antigen-presenting cells (APCs) to present viral or tumor antigens to reactive T cells and amplifies Th1-mediated immune response [24, 26]. Because there are a number of cell types that express either TLR7 or receptors for cytokines induced by imiquimod, this agent has broad-reaching, direct and indirect effects in the skin as well as the related skin immune system [24]. Thus, imiquimod has been demonstrated to be useful in the treatment of a number of conditions beyond the FDA-approved indications [24]. However, the effect of TLR7 on Treg cell function in cancer immunosurveillance is still not well understood [27, 28].

In this study, we found that Loxoribin, one of the TLR7 ligands, could inhibit tumor growth *in vivo* in both colon cancer and lung cancer xenograft models, and these antitumor effects of Loxoribin were mediated by promoting CD4^+^T cell proliferation and reversing Treg-mediated suppression via DCs. However, deprivation of IL-6 using a neutralizing antibody abrogated the ability of DCs to reverse the Treg cell-mediated suppression, restoring CD4^+^CD25^−^T cell proliferation to near normal levels. Furthermore, adoptive transfer of Loxoribin-treated DCs inhibited the tumor growth *in vivo*. Therefore, this study links TLR7 signaling to the functional control of Treg cells and identifies Loxoribin as a new therapeutic strategy in cancer treatment, which may offer new opportunities to improve the outcome of cancer immunotherapy by administration of TLR7 agonist.

## RESULTS

### TLR7 ligand Loxoribin inhibits tumor growth *in vivo*

**Figure 1:**
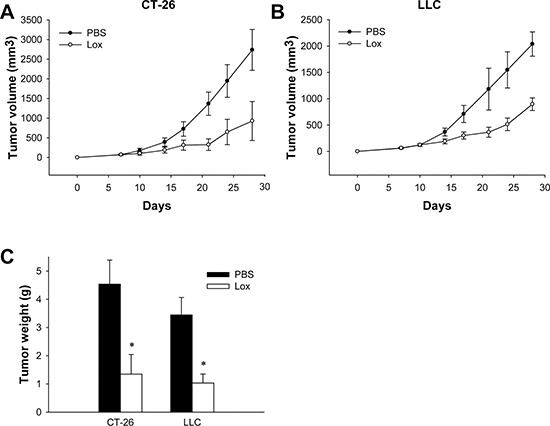
TLR7 ligand Loxoribin inhibits tumor growth *in vivo* **(A–B)** CT-26 and LLC cells were transplanted into mice (*N* = 5 per group). Tumor size was measured twice a week for indicated period. The growth curves of tumor are shown. **(C)** Average weight of tumors of each group (*N* = 5). Data are representative of three independent experiments. *P < 0.05.

To determine the role of TLR7 in cancer, we initially investigated its effect on two tumor models *in vivo*, i.e. a colorectal cancer model and a Lewis Lung Cancer (LLC) tumor model. CT-26 colon cancer cells and LLC Lewis lung cancer cells were subcutaneously (s.c) injected into mice respectively, and tumor size was monitored. Seven days later, when tumors were palpable, mice were then intraperitonealy (i.p) injected with Loxoribin, which were repeated twice a week. As shown in Figure 1A and Figure 1B, CT-26 cells and LLC cells showed progressive growth but were inhibited by Loxoribin. After 28 days, mice were euthanized and the tumor weights were measured. The average weight of the CT-26-tumors or LLC-tumors was significantly less than that of the Loxoribin-treated counterparts (Figure 1C, *P* < 0.01). These results indicate that TLR7 ligand Loxoribin inhibits tumor growth *in vivo*.

### The anti-tumor effect of Loxoribin is elicited by rendering CD4^+^CD25^−^T cells refractory to the suppressive effect of Treg cells

**Figure 2:**
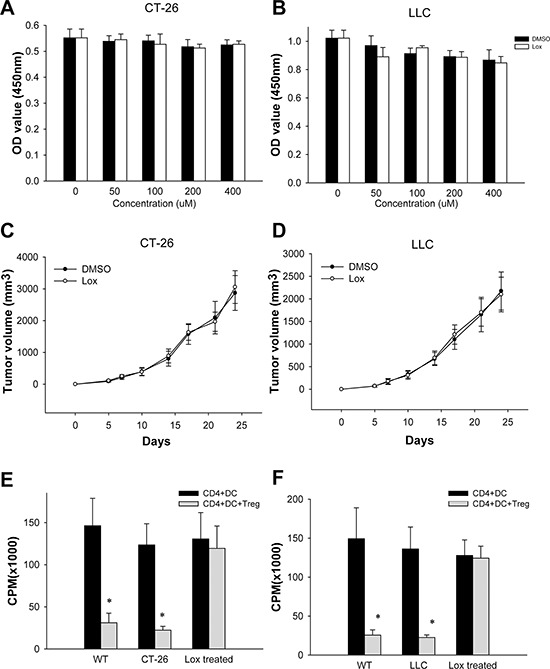
The antitumor effect of Loxoribin is elicited by rendering CD4^+^CD25^−^T cells refractory to the suppressive effect of Treg cells **(A–B)** CT-26 and LLC cells were stimulated with Loxribine for 48 hours, and the effect of Loxribine on cell proliferation was measured by CCK-8 assay. **(C–D)** CT-26 and LLC cells were transplanted into SCID mice (*N* = 5 per group). Tumor size was measured twice a week for indicated period. The growth curves of tumor are shown. **(E–F)** Naïve CD4^+^CD25^−^T cells, CD4^+^CD25^+^T (Treg) cells and DCs were purified by magnetic-activated cell sorting from wild type mice and tumor-bearing mice. CD4^+^CD25^−^T cells and CD4^+^CD25^+^Treg cells were co-cultured with irradiated DCs in anti-CD3/anti-CD28 coated plate. The effect of Loxribine on the suppressive functions of Tregs was assayed by incorporation of tritiated thymidine. Data are representative of three independent experiments. *P < 0.05.

We next investigated the mechanism behind the anti-tumor effect of Loxoribin. To define whether Loxoribin has a direct tumoricidal effect on CT-26 cells, we first detected the expression of TLR7 in CT-26 and LLC cells. No TLR7 expression was detected, using RT-PCR, in CT-26 and LLC cells (data not shown). In a WST assay, Loxoribin treatment did not affect CT26 and LLC cell proliferation, indicating that the anti-tumor effect of Loxoribin is not mediated by its direct tumoricidal activity (Figure 2A–2B). To further determine whether Loxoribin activates innate immune cells to induce tumor remission, we inoculated CT-26 and LLC cells into SCID mice that have an intact innate system but lack T or B cells. When tumors were palpable, mice were i.p. injected with Loxoribin twice a week. CT-26 and LLC cells grew progressively in SCID mice, and Loxoribin treatment did not inhibit the tumor growth (Figure 2C–2D), indicating that the antitumor effect of Loxoribin is not elicited via its innate immune cell activation either. To investigate whether TLR7 ligand has an effect on the suppressive functions of Tregs, we next purified naïve CD4^+^CD25^−^T cells, CD4^+^CD25^+^ (regulatory) T cells and DCs by magnetic-activated cell sorting from wild type mice and tumor-bearing mice. Then, CD4^+^CD25^−^T cells and CD4^+^CD25^+^Treg cells were co-cultured with irradiated DCs in anti-CD3/anti-CD28 coated plate. We found that Tregs from both wild type and tumor-bearing mice profoundly suppressed CD4^+^CD25^−^T cell proliferation as assayed by incorporation of tritiated thymidine (Figure 2E–2F). However, Tregs from both Loxoribin-treated tumor-bearing mice failed to suppress the CD4^+^CD25^−^T cell proliferation. Thus, the anti-tumor effect of Loxoribin is elicited via rendering CD4^+^CD25^−^T cells refractory to the suppressive effect of Treg cells.

### Ligation of TLR7 onto DCs promotes CD4^+^T cells proliferation

**Figure 3:**
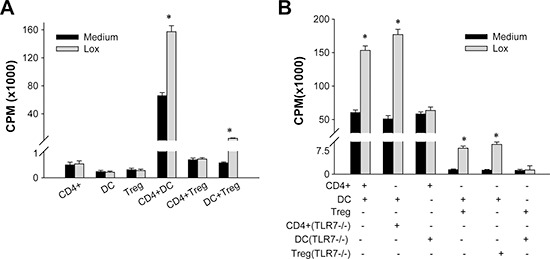
Ligation of TLR7 on DCs promotes CD4^+^T cells proliferation **(A)** Naïve CD4^+^CD25^−^T cells, CD4^+^CD25^+^Treg cells or DCs were first cultured in the presence or absence of TLR7 ligand Loxoribin, and the direct effect of Loxoribin on the T cell proliferation ex vivo was determined by incorporation of tritiated thymidine. Then, CD4^+^CD25^−^T cells or CD4^+^CD25^+^Tregs co-cultured with DCs in the presence of Loxoribin, and the effect of Loxoribin on T cell proliferation was determined. **(B)** CD4^+^CD25^−^T cells, CD4^+^CD25^+^Treg or DCs were purified from both wild-type and TLR7−/− mice, and then co-cultured with different combinations of either wild-type or TLR7−/− T cell subsets in the presence of wild-type or irradiated TLR7−/−DCs, the effect of Loxoribin on the T cell proliferation was determined by incorporation of tritiated thymidine. Data are representative of three independent experiments. *P < 0.05.

To investigate how TLR7 activation by Loxoribin renders CD4^+^T cells refractory to the suppressive effect of Treg cells, we first determined the direct effect of Loxoribin on the T cell proliferation ex vivo. Naïve CD4^+^CD25^−^T cells, CD4^+^CD25^+^Treg cells or DCs were first cultured in the presence or absence of Loxoribin, respectively. As shown in Figure 3A, Loxoribin did not directly induce proliferation of CD4^+^CD25^−^T cells, CD4^+^CD25^+^Treg cells or DCs. We further determined whether this effect was dependent on the cell-cell communication, as shown in Figure 3B, a significantly increased proliferation was observed in CD4^+^CD25^−^T cells or CD4^+^CD25^+^Tregs co-cultured with DCs in the presence of Loxoribin, while addition of Loxoribin had no effect on proliferation when CD4^+^CD25^−^T cells and CD4^+^CD25^+^Treg cells were co-cultured together. These results suggest that Loxoribin may function on DCs to promote CD4^+^CD25^−^T cell and CD4^+^CD25^+^Treg proliferation. To verify this possibility, CD4^+^CD25^−^T cells, CD4^+^CD25^+^Tregs or DCs were purified from both wild-type and TLR7−/− mice, and co-cultured with different combinations of either wild-type or TLR7−/− T cell subsets in the presence of irradiated TLR7−/− DCs. We found that deficiency of TLR7 in the CD4^+^CD25^−^T cells or CD4^+^CD25^+^Treg cells did not affect the promotion function of Loxoribin on the proliferation of CD4^+^CD25^−^Tcells or CD4^+^CD25^+^Treg cells (Figure 3B). However, when CD4^+^CD25^−^T cells or CD4^+^CD25^+^Treg cells were combined with TLR7−/− DCs, Loxoribin had no observable effects on cell proliferation (Figure 3B). Collectively, these results indicate that the proliferation-promoting function of Loxoribin on CD4^+^CD25^−^T cells or CD4^+^CD25^+^Tregs is meditated by DCs.

### Ligation of TLR7 onto DCs reverses Treg cell-mediated suppression

**Figure 4:**
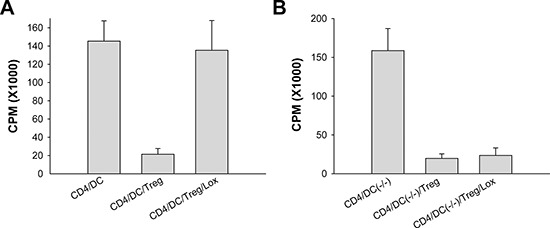
Ligation of TLR7 on DCs reverses CD4^+^CD25^+^Treg cell-mediated suppression CD4^+^CD25^+^Tregs and naive CD4^+^CD25^−^T cell subsets were purified and co-cultured with irradiated wild-type **(A)** or TLR7−/−DCs **(B)** in a conventional suppression system, the effect of Loxoribin on the surpressive function of Tregs was determined by incorporation of tritiated thymidine.

Given that ligation of TLR7 onto DCs promotes CD4^+^T cell proliferation and renders these responder T cells refractory to the suppression of Tregs, the other possibility is that Loxoribin-treated DCs may directly reverse Treg-mediated suppressive function. We therefore determined whether Loxoribin-treated DCs could break Treg-mediated suppression. CD4^+^CD25^+^Tregs and naive CD4^+^CD25^−^T cell subsets were purified and co-cultured with irradiated DCs in a conventional suppression system. Addition of Loxoribin into the conventional suppression system abrogated Treg cell-mediated suppression, restoring CD4^+^CD25^−^T cell proliferation to a near-normal level (Figure 4A). To further determine the significance of TLR7 expression in DCs in Loxoribin-triggered antitumor immunity, CD4^+^CD25^−^T cells, CD4^+^CD25^+^Tregs or DCs were purified from both wild-type and TLR7−/− mice respectively, and then co-cultured with different combinations in the presence of or absence of Loxoribin. As expected, Loxoribin could not reverse the suppressive function of Treg cells when TLR7 expression was specifically deficient in DCs (Figure 4B). Taken together, these results suggest that ligation of TLR7 onto DCs is important for abrogating CD4^+^CD25^+^Treg cell-mediated suppression.

### IL-6 secreted by DCs is critical for abrogating suppression

**Figure 5:**
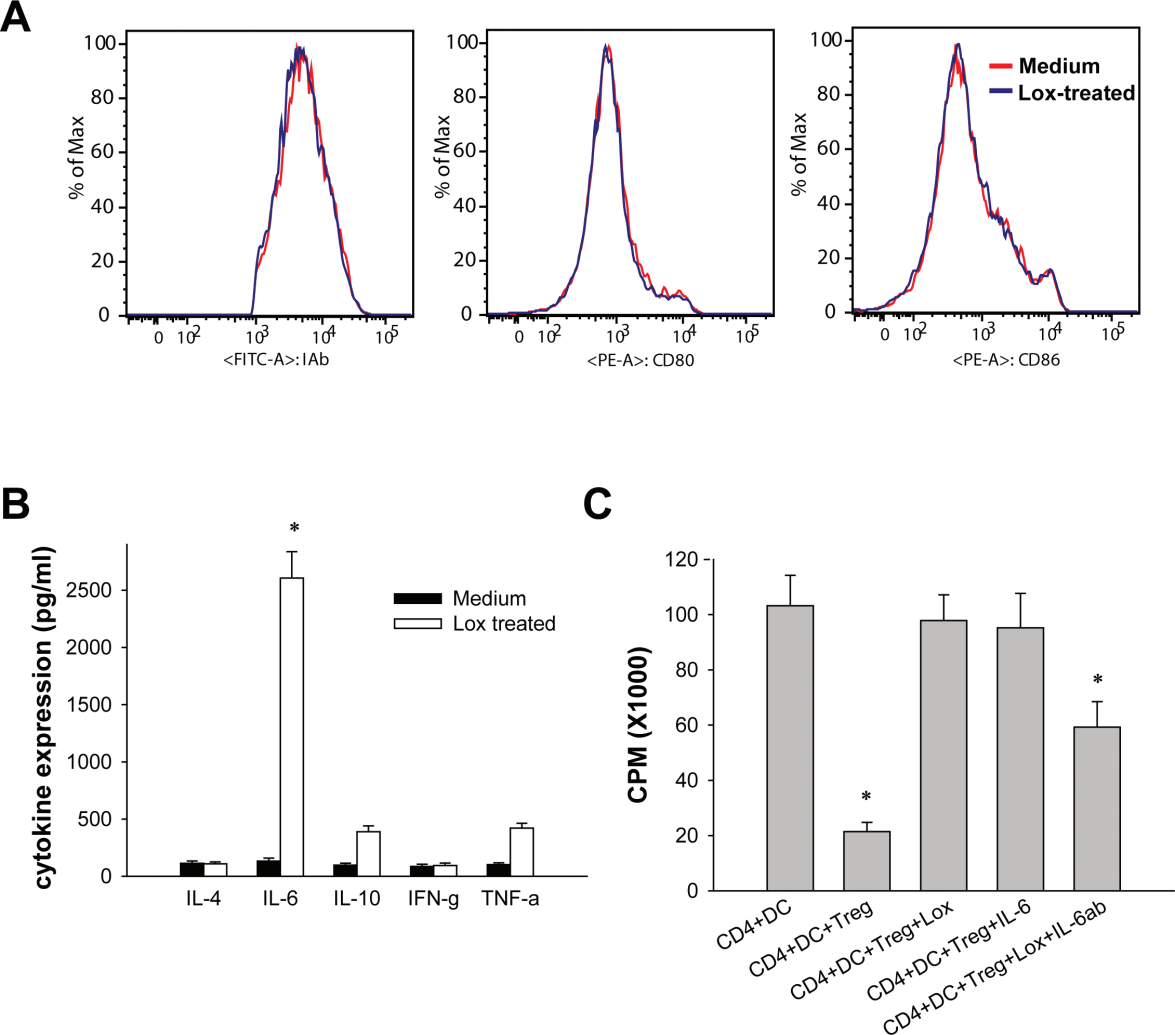
IL-6 secreted by DCs is critical for abrogating suppression **(A)** After stimulation with Loxoribin, the expression of CD80, CD86 and MHC II on DCs were determined by flow cytometry analysis. **(B)** The cytokine production by Loxoribin-treated DCs was quantified by ELISA. **(C)** CD4^+^CD25^−^T cells were cultured with CD4^+^CD25^+^T cells in the presence or absence of TLR7 ligand Loxoribin, IL-6 or neutralizing IL-6 antibody. After 56 h of culture, the cell proliferation was measured by the incorporation of tritiated thymidine. Data are representative of three independent experiments. *P < 0.05.

To define how DCs regulated Treg-mediated suppression when TLR7 was activated, we first detected the maturation of DCs after Loxoribin treatment. We found that expression of CD80, CD86 and MHC II in DCs were not affected by Loxoribin (Figure 5A), which suggest that Loxoribin does not affect the maturation of DCs. Then we determined whether these effects needed cell-cell contact. Loxoribin-treated DCs were co-cultured with CD4^+^CD25^−^T cells and CD4^+^CD25^+^Tregs in a trans-well culture system. As shown in Figure 5, Loxoribin-treated DCs reversed the Treg cell-mediated suppression, restoring CD4^+^CD25^−^T cell proliferation to a near-normal level, suggesting that one or some cytokines secreted by Loxoribin-stimulated DCs act on CD4^+^CD25^−^T cells and make them refractory to the suppression of Treg cells. To explore which soluble factor or factors is or are pivotal to this refractoriness, we measured the cytokine production of Loxoribin-treated DCs by ELISA. We found that although TNF-α and IL-10 were higher in Loxoribin-treated DCs culture supernatant, increase of IL-6 was much dramatic (Figure 5B). We next added IL-6 instead of Loxoribin into the culture system, and found that the IL-6 reversed the suppressive function of Tregs. However, neutralization of IL-6 with IL-6 neutralizing antibody abrogated the ability of DCs to reverse suppression (Figure 5C). Therefore, IL-6 plays a critical role in the TLR7-mediated block of suppression of Tregs by DCs.

### Loxoribin-treated DCs inhibit tumor growth *in vivo*

**Figure 6:**
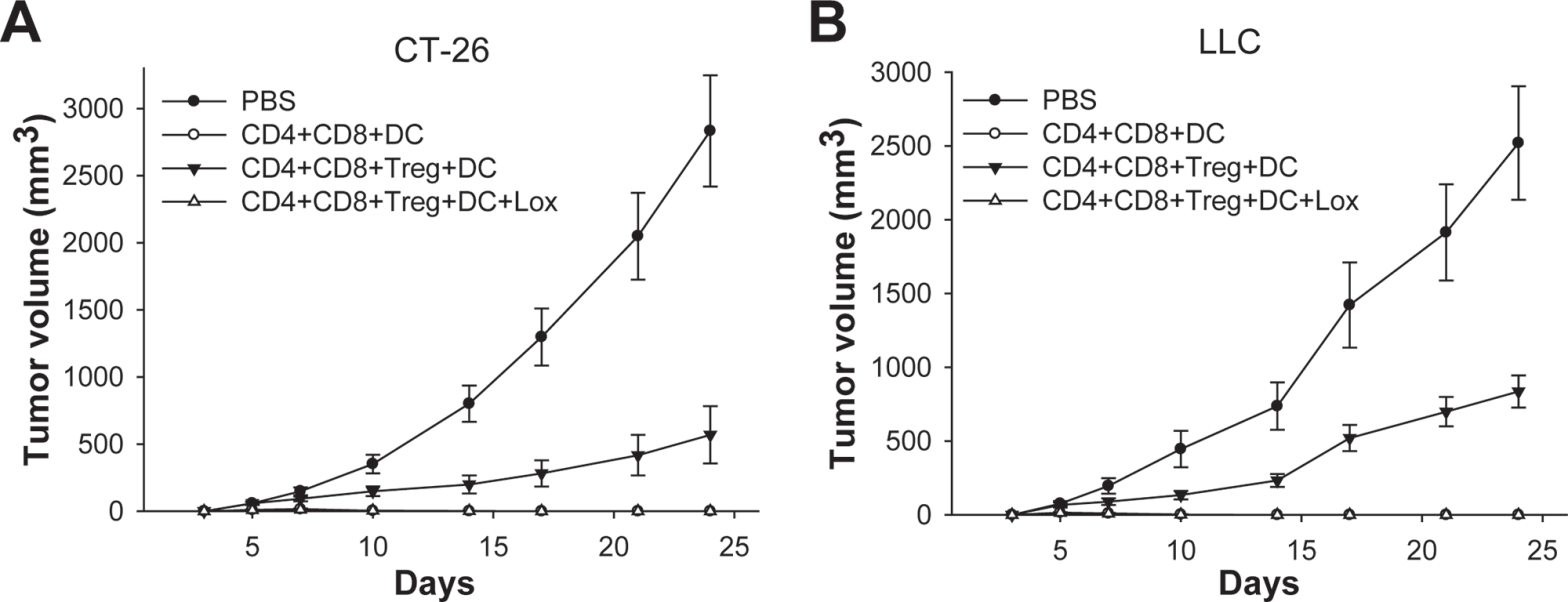
Loxoribin-treated DCs inhibit tumor growth *in vivo* BALB/c or C57BL/6 mice were s.c. injected with CT-26 **(A)** or LLC **(B)** tumor cells (*N* = 5 per group). Five days after tumor cell inoculation, CD4^+^CD8^−^T cells, CD4^−^CD8^+^T cells, and CD4^+^CD25^+^Tregs were mixed with Loxoribin-treated or non-treated DCs and then injected i.v into tumor cell transplanted mice. Tumor growth was monitored twice a week. Data are representative of three independent experiments.

To determine the importance of TLR7 expression in DCs in Loxoribin-triggered antitumor immunity *in vivo*, we purified CD4^+^CD8^−^T cells, CD4^−^CD8^+^T cells and CD4^+^CD25^+^Treg cells, and mixed them with Loxoribin-treated or non-treated DCs from wild type mice as indicated, and then injected them into tumor cell bearing mice. Tumor size was monitored twice a week. As showed in Figure 6, we found that CT-26 cells (Figure 6A) and LLC cells (Figure 6B) showed progressive growth but were inhibited when co-injected with autologous CD4^+^CD8^−^T cells or CD4^−^CD8^+^T cells. However, when CD4^+^CD25^+^Tregs were adoptively transferred into mice, tumor cells again grew progressively and more rapidly than in mice receiving CD4^+^CD8^−^ or CD4^−^CD8^+^T cells alone. In contrast, tumor growth was significantly inhibited in mice adoptive transferred with Loxoribin-treated DCs, compared with that in mice adoptive transferred with Tregs and CD4^+^CD8^−^ or CD4^−^CD8^+^T cells (Figure 6A–6B). These suggest that Loxoribin-treated DCs can impair Tregs' suppressive function and enhance the killing ability of CD4 /CD8 to tumor *in vivo*.

## DISCUSSION

In this study, we have demonstrated that i.p administration of the TLR7 agonist Loxoribin leads to tumor regression in two tumor models, i.e. a colorectal cancer model and a LLC tumor model. TLRs are important molecules in the regulation of innate and adaptive immunity. TLR7 agonists have been shown to promote DCs maturation, improve T-cell priming and activate innate immune cells such as NK and NKT cells [25, 29–31]. However, the precise mechanism by which Loxoribin induces the antitumor response is unclear. Here, we found that CT-26 and LLC cells did not express TLR7, and the Loxoribin-mediated antitumor effect did not include a direct tumoricidal activity, nor did it activate innate immune cells, suggesting a mechanism of action involved in host adaptive immune responses.

Treg cells are increased in cancer and are an obstacle for immune surveillance and immune therapy of cancer [32, 33]. Approaches that aim to break through such an obstacle would be beneficial to the treatment of cancer patients, by means of eliminating CD4^+^CD25^+^Treg cells with a specific antibody [34, 35]. However, this approach may not efficiently eliminate Treg cells, and it depletes on both Treg cells and activated effector cells, as the CD25 marker is positive in both Treg cells and all activated T cells [36, 37]. Recent findings have demonstrated that TLRs and their ligands play important roles in the regulation of suppressive function of Tregs [38, 39]. LPS, the ligand of TLR4, can interact with TLR4 expressed by DCs and subsequently activate MyD88 signal pathway to release the suppressive function of Tregs on conventional CD4^+^T cells [38, 40, 41]. Both TLR2 ligand Pam3Cys-SK4 and TLR8 ligand Poly(G) can directly act on Tregs and reverse its suppressive function on CD4^+^T cells [23, 42, 43]. In the present study, we show that TLR7 activation by Loxoribin induces tumor regression *in vivo*, and these anti-tumor effects are mediated by promoting CD4^+^T cell proliferation and reversing Treg-mediated suppression. The findings presented here uncover a novel mechanism linking TLR7 signaling to the modulation of effector cells and the Tregs' function.

TLRs control activation of the innate and adaptive immune responses, which is generally produced by inducing the maturation of DCs [44, 45]. How Loxoribin signals regulate the suppressive function of Treg cells remains unknown. In this study, we found that Loxoribin could promote the proliferation of CD4^+^T cells and made Tregs lose the suppressive function only in the presence of DCs. However, these effects of Loxoribin were mediated neither by induction of DCs maturation nor by direct cell-cell contact. Previous study has shown that TLR stimulation, such as LPS, and CpG, induces DCs to secrete cytokines, including IL-6, that render CD4^+^ effector cells refractory to Treg cell-mediated suppression [20, 41, 46]. Bourquin et al have reported that an agonist of TLR7 may also promote antitumor T-cell responses through the induction of multiple type-1 cytokines such as IFNα, IFNγ and IL-12 [47, 48]. A recent report has also shown that activation of splenic DCs by TLR7 ligand decreases the number of Tregs, leading to a reduced and less-stable Foxp3 expression and impaired suppressive function [49]. Foxp3 plays important role in the suppressive function of regulatory T cells. In our study, we have tried to verify whether the expression of Foxp3 was changed followed by TLR7 ligand Loxoribin treatment by using Foxp3-GFP report mice. Unfortunately, we found that Tregs cultured with untreated DCs or Tregs cultured alone also lose some expression of Foxp3 after 3 days *in vitro* culture. We are trying to figure out why the Foxp3 is not stable *in vitro*, and did not present these data in the current manuscript. In our study, we shown that TLR7 ligand Loxoribin triggered stronger IL-6 response in DC/T/Treg cells co-culture, the loss of suppressive function of Treg cells caused by Loxoribin in the co-culture system could be reproduced by supernatants of Loxoribin-treated DCs or IL-6, but not by supernatants of untreated or Loxoribin-treated DCs that had been pretreated with IL-6 neutralizing antibody against IL-6. Although other potential mechanisms that mediate the Loxoribin-mediated anti-tumor effects cannot be excluded, our results suggest that IL-6 produced by Loxoribin-treated DCs in the co-culture system is largely responsible for the reversal of Treg-mediated suppression.

Taken together, Loxoribin treatment leads to tumor regression, and the underlying mechanism involves modulation of the CD4^+^T cell proliferation and the suppressive activity of Tregs via DCs in a TLR7-dependent manner. In the light of our observation of the therapeutic potential of Loxoribin in colon cancer and lung cancer, TLR7 therapy may be important for human cancer, and TLR7 ligands and their analogs could be immunotherapeutic modalities in cancer patients in the future.

## MATERIAL AND METHODS

### Mice, tumor cell lines and reagents

BALB/c, C57BL/6 and SCID mice were purchased from the Shanghai SLAC Laboratory Animal Company, China. TLR7−/− mice were purchased from the Jackson Laboratory (Bar Harbor, ME). All animals were maintained under specific pathogen-free conditions in the animal facilities of Shanghai Jiaotong University, School of Medicine. The murine colon cancer cell line CT-26 and the Lewis lung carcinoma tumor cell line LLC were purchased from Shanghai Institutes for Biological Sciences, Chinese Academy of Sciences. Tumor cells were cultured in complete RPMI 1640 medium supplemented with 10% FBS, 100 U/ml penicillin and 100 g/ml streptomycin in a humidified cell incubator with an atmosphere of 5% CO_2_ at 37°C. Exponentially growing cells were used for experiments. TLR7 ligand Loxoribin was purchased from Invivogene (San Diego, CA, USA).

### Quantitative PCR

Total RNA was extracted using Trizol reagent (Invitrogen, Carlsbad, CA, USA) following the manufacturer's manual. RNA (1 μg) was reversely transcribed to cDNA using Reverse Transcription system (Promega, Madison, WI, USA). QRT-PCR was performed to quantify the mRNA levels of TLR7 with the SYBR Green PCR core Reagent kit (Applied Biosystems, Foster City, CA, USA). GAPDH was used as the endogenous reference. Data were analyzed by using the comparative Ct method. Specificity of resulting PCR products was confirmed by melting curves. The primers used in this assay were: TLR7 forward: TGACTCTCTTCTCCTCCA, TLR7 reverse: GCTTCCAGGTCTAATCTG; GAPDH forward: CACCCTTCAAGTGGGCCCCG, GAPDH reverse: TCCAGGAGCGAGACCCCACT.

### Cell isolation

Murine CD11c^+^DCs, CD4^+^CD25^+^Tregs and CD4^+^CD25^−^T cells were purified from spleen and lymph nodes by MACS according to the manufacturer's instructions (Miltenyi Biotec). The purity of the isolated T and DC cell subpopulations was >95% as assessed by flow cytometry analysis.

### Tumor cell proliferation assay

Cell proliferation was assessed by WST (water-soluble tetrazolium salt) assay using a Cell Counting Kit-8 (Dojindo, Kumamoto, Japan) according to the manufacturer's instructions. Tumor cells (2 × 10^3^ cells/well) were seeded in 96-well plates and treated with the TLR7 ligand Loxoribin. The plates were incubated for 5 days. The number of viable cells was assessed by measurement of the absorbance at 450 nm.

### T cell proliferation/Treg suppression assay

CD4^+^CD25^−^T cells (1 × 10^5^) were cultured in U-bottom 96 well plates with 10^4^ splenic DCs, 100 ng/ml anti-CD3, 100 ng/ml anti-CD28mAb and CD4^+^CD25^+^T cells at different ratios (1: 0.5, 1:0.2 and 1:0.1) in the presence or absence of Loxoribin, 100 ng/ml IL-6 (PeproTech, Rocky Hill, NJ, USA) or neutralizing IL-6 antibody (5, 10, 20 μg/ml) (R&D Systems, Minneapolis, MN, USA). For T cell proliferation assay without DCs, 1 × 10^5^ CD4^+^CD25^−^T cells were cultured with CD4^+^CD25^+^T cells at different ratios, 2.5 μg/ml anti-CD28 mAb in anti-CD3 mAb-coated (5 μg/ml) 96-well plates in the presence or absence of Loxoribin, IL-6 (100 ng/ml) or neutralizing IL-6 antibody. After 56 h of culture, (^3^H) thymidine was added at a final concentration of 1 μCi/well, followed by an additional 16 h of culture. The incorporation of (^3^H) thymidine was measured with a liquid scintillation counter. All experiments were performed in triplicate.

### Cytokine ELISA

The cytokine concentrations in serum and culture supernatant were quantified by ELISA according to the manufacturer's instructions (R&D Systems, Minneapolis, MN, USA).

### Tumour xenograft model and tumorigenicity assay

BALB/c, C57BL/6 or SCID mice were s.c. injected with CT-26 or LLC tumor cells. Tumor growth was monitored twice a week, and tumor volume was assessed by measuring tumor size with digital calipers using the following formula: volume = ab^2^π/6. For treatment, 5 days after tumor cell inoculation, mice were administered i.p with Loxoribin (400 μg/mouse) or PBS twice a week. In the experiment of DCs treatment, BALB/c or C57BL/6 mice were s.c. injected with CT-26 or LLC tumor cells. Five days after tumor cell inoculation, CD4^+^CD8^−^T cells, CD4^−^CD8^+^T cells and CD4^+^CD25^+^Tregs were mixed with Loxoribin-treated or non-treated DCs and then i.v. injected into tumor cell bearing mice. Tumor growth was monitored weekly and tumor volume was assessed.

### Statistical analysis

Results were summarized as means ± SEM. Student *t* test and one-way analysis of variance (ANOVA) were used to analyze the data and the significance level was set at *P* < 0.05.
